# Resolutions on the Death of Geo. H. Cushing, M.D., D.D.S., Chicago, Ills.

**Published:** 1900-06-15

**Authors:** A. W. Harlan, Truman W. Brophy, Joseph W. Wassall

**Affiliations:** Committee.; Committee.; Committee.


### RESOLUTIONS ON THE DEATH OF GEO. H. CUSHING, M.D., D.D.S., CHICAGO, ILLS.

It has come to the notice of this society that our beloved brother and fellow practitioner, Geo. H. Cushing, one of the founders of this society, departed this life May 25, 1900, at Los Angeles, California; therefore, be it

*Resolved*, That we mourn his loss as personal, because every member of the society knew him and loved him as a friend, counsellor and guide. This is no ordinary expression from a committee appointed to draw a memorial, but one where every member feels that it is a personal loss to no longer see and feel the presence of one much beloved; be it further

*Resolved*, That in Dr. Cushing we mourn the loss of one who was in every sense an inspiration to do one's best in all the complex hours of life's duties. He was monitor, teacher and helper in all emergencies. His life was devoted to his profession in a sense little understood by the thoughtless, and his influence for good was far reaching and always to be relied upon.

We feel that in this feeble tribute to the vast labors he performed for forty years, that we fell short in our estimation of them, because no one was more staunch in his devotion to public or private duties than our departed friend. He had his faults, but they were so obscured by his noble and gentle nature that few knew of them, so they must lie in an oblivion so perfect that only his most bitter enemy may recall them. For us he was a pattern of the kindly professional gentleman and devoted friend.

We extend to his family our condolence and this expression of our sorrow. We will all go to his last resting place feeling that life is only a brief day, and we will meet him again in the near future, where all is peace and serenity.

*Resolved*, That a copy of this tribute be sent to the family of the deceased and to the dental journals for publication.

A. W. Harlan,

Truman W. Brophy,

Joseph W. Wassall, } *Committee.*

